# Unraveling the Molecular Landscape of Follicular Fluid: A Comparative Proteomic Study Between Bali and Brahman Cattle

**DOI:** 10.1155/vmi/5512313

**Published:** 2026-08-04

**Authors:** Sri Gustina, Hasbi Hasbi, Ismah Ulfiyah Azis, Tulus Maulana, Siti Nuraisya Hamsir

**Affiliations:** ^1^ Department of Animal Production, Faculty of Animal Science, Hasanuddin University, Jl. Perintis Kemerdekaan Km. 10 Tamalanrea, Makassar 90245, South Sulawesi, Indonesia, unhas.ac.id; ^2^ Department of Animal Nutrition, Faculty of Animal Science, Hasanuddin University, Jl. Perintis Kemerdekaan Km. 10 Tamalanrea, Makassar 90245, South Sulawesi, Indonesia, unhas.ac.id; ^3^ Research Center for Applied Zoology, National Research and Innovation Agency, Jl. Raya Bogor Km. 46, Cibinong 16911, Indonesia, brin.go.id; ^4^ Magister Programme in Animal Science and Technology, Faculty of Animal Science, Hasanuddin University, Jl. Perintis Kemerdekaan Km. 10 Tamalanrea, Makassar 90245, South Sulawesi, Indonesia, unhas.ac.id

**Keywords:** Bali cattle, biomarker proteins, Brahman cows, follicular fluid, proteomic

## Abstract

**Background:**

Protein biomarkers are increasingly recognized as indicators of metabolic status, immune response, and reproductive performance in cattle. However, comparative data among different breeds commonly raised in Indonesia remain limited.

**Objectives:**

This study aimed to compare the specific proteins in follicular fluid (FF) of Bali and Brahman cows.

**Methods:**

Samples from FF (*n* = 20 pairs of ovarium) were analyzed for identification of specific proteins using LC–HRMS analysis.

**Results:**

Proteomic analysis revealed distinct breed‐specific FF profiles, with 16 proteins uniquely identified in Bali cattle and 23 proteins in Brahman. Bali FF was enriched with proteins associated with protease inhibition, coagulation regulation, extracellular matrix remodeling, and cytoskeletal dynamics, including CORO1C, ZER1, YARS1, and L3MBTL3. In contrast, Brahman FF showed enrichment of proteins involved in lipid oxidation, one‐carbon metabolism, and peroxisomal activity, notably ACOX2, BHMT, and KIAA0586.

**Conclusion:**

Here, we present the proteomic characterization of FF from Bali and Brahman cattle, where several bioactive proteins are candidate proteins potentially associated with reproductive processes. These findings highlight the potential of protein biomarkers in guiding cattle breeding and management strategies in tropical regions.

## 1. Introduction

Follicular fluid (FF) is a complex biological medium that plays a crucial role in oocyte development and follicular maturation in mammals. The molecular composition of FF reflects the metabolic, physiological, and endocrine status of the ovary, serving as an important indicator of oocyte competence and fertility [1]. Proteomic studies of FF have become a strategic approach to understanding the molecular regulation of reproductive processes, as the proteins within FF function as mediators of communication between granulosa cells, theca cells, and the oocyte [2, 3].

The proteomic composition of FF is known to vary among species and even among cattle breeds, reflecting genetic adaptations to environmental conditions, diet, and reproductive selection [4, 5]. Bali cattle (*Bos javanicus*) and Brahman cattle (*Bos indicus*) exhibit significant genetic and physiological differences. Bali cattle are known for their high tolerance to heat stress, metabolic efficiency, and relatively good fertility under tropical conditions [6], whereas Brahman cattle are more oriented toward beef production performance and display distinct reproductive responses to environmental stress [7]. Comparative studies between these two breeds remain very limited at the molecular level, particularly in the context of the proteomic profile of FF. Given the genetic and physiological adaptations distinguishing Bali and Brahman cattle, an in‐depth analysis of the FF proteomic landscape has the potential to reveal molecular biomarkers that explain variations in reproductive performance between breeds [8, 9].

Shotgun proteomics or label‐free quantitative proteomics approaches using LC–MS/MS technology have been applied to identify proteins involved in oogenesis, steroidogenesis, and oocyte maturation [10]. Several proteins such as albumin, transferrin, and apolipoprotein have been identified as key regulators of the follicular microenvironment [11, 12]. In addition, proteins related to oxidative stress, immunomodulation, and growth factors (e.g., IGFBP, HSP70, and SOD) are often associated with oocyte quality and fertility [13–15].

Most proteomic studies on FF have focused solely on *Bos taurus*, whereas research involving Bali cattle and Brahman cattle remains scarce. The lack of molecular data limits the understanding of the biological basis underlying fertility differences among tropical cattle breeds. Therefore, a comparative proteomic exploration of FF between these two breeds is expected to identify potential biomarkers related to oocyte quality and fertility level, while also providing a molecular foundation for genetic selection programs and the improvement of reproductive efficiency in Indonesian local cattle.

## 2. Materials and Methods

### 2.1. Materials

A total of 20 ovarium pairs in luteal phase (each of 10 pairs from Bali and Brahman cows as replicates) were collected from the Tamangapa Slaughterhouse to laboratory for one hour in collection medium. The animals used in this study were around 5–8 years old with a parity of 2‐4 births. Preparation and collection of FF were aspirated from secondary stage at the In Vitro Embryo Production Laboratory, Hasanuddin University. Proteomic testing and specific protein characterization were conducted at the PT. Wiralab Analitika Solusindo, DIY Yogyakarta.

### 2.2. Ethical Approval

All procedures performed in this study were reviewed and approved by the Ethics Committee on the Use of Research and Learning Animals of the Faculty of Animal Science, Hasanuddin University (approval no. 026/UN4.12/EC/IX/2025).

### 2.3. Methods

A. Collection of ovarium and FF samples
 Ovaries were transported from the slaughterhouse to the laboratory using 0.9% NaCl media supplemented with 100 IU/mL penicillin and 100 μg/mL streptomycin sulfate. FF was then collected from each pair of ovaries using an 18G needle and then centrifuged for 15 min at 3000 × *g*, and the fluids were placed in Eppendorf tubes for storage at −80°C before analysis [4].
B. Characterization of specific proteins in FF

### 2.4. Proteomic Sample Preparation

A total of 500 μL FF was transferred into microcentrifuge tubes, followed by the addition of 1 mL of prechilled acetone. The mixture was incubated for 1 h at −20°C to allow protein precipitation. Samples were then centrifuged at 3000 × *g* (Eppendorf, Hamburg, Germany) for 10 min at 4°C, after which the supernatant was discarded. The resulting pellet was washed three times with cold acetone and subjected to gentle centrifuge‐assisted evaporation until nearly dry. The dried pellet was resuspended in 500 μL of 50 mM ammonium bicarbonate (Fisher Chemical, USA). Protein reduction was performed by adding 10 μL of 50 mM dithiothreitol (Merck, Germany) and incubating the mixture at 55°C for 60 min. Subsequently, alkylation was carried out by adding 10 μL of 50 mM iodoacetamide (Merck, Germany) and incubating the samples at room temperature in the dark for 60 min. Proteolytic digestion was performed using mass spectrometry–grade trypsin (Pierce Thermo, Illinois, USA) at an enzyme‐to‐substrate ratio of 1:50 (w/w), followed by incubation for 17 h at 37°C (modified from Dongiovanni et al.) [16]. Digested samples were acidified with 500 μL of 1% trifluoroacetic acid (TFA) and centrifuged at 12,000 × *g* (Eppendorf, Hamburg, Germany) for 10 min at 4°C. The clarified peptide solution was passed through a 0.2 μm PVDF filter (Thermo Scientific, USA) before being transferred to autosampler vials and prepared for LC–HRMS injection.

The proteomic analysis of FF was conducted using an LC–HRMS assay with liquid chromatography (Thermo Scientific Vanquish Horizon UHPLC with Binary Pump, Germering, Germany) and high‐resolution mass spectrometry (Thermo Scientific Orbitrap Exploris 240 HRMS, Bremen, Germany). Peptide separation was achieved using a mobile phase of MS‐grade water containing 0.1% formic acid (A) and MS‐grade acetonitrile containing 0.1% formic acid (B), employing a gradient technique. The gradient started with 5% B for 1 min, increased to 50% B buffer over 30 min, maintained at 50% B for 2 min, then increased to 90% B for 2 min, and finally returned to 5% B for 45 min at a flow rate of 75 μL/min. The analysis used a Thermo Scientific Acclaim Acclaim PepMap 100 C18 (150 mm length × 1 mm ID × 3 μm particle size) (Lithuania) at 30°C with a 10 μL injection volume. Mass spectrometry was performed on a Thermo Scientific Orbitrap Exploris 240 HRMS (Bremen, Germany) in full MS/dd‐MS2 mode with positive polarity, a sheath gas flow of 10 AU, and an auxiliary gas flow of 5 AU. The ion transfer tube temperature was set to 300°C with a spray voltage of 4000 V. The scanning range was from 350 to 1200 m/z, with a full MS resolution of 120,000 FWHM and 15,000 FWHM for dd‐MS2 (Mergner et al. and Windarsih et al. with modifications) [17, 18].

### 2.5. Database, Bioinformatics Analysis, and Pathway Analysis

All data were obtained and processed using the UniProt bovine protein database (https://www.uniprot.org) [19], a resource provided by the European Molecular Biology Laboratory (EMBL) Incorporated Association, and then cross‐referenced with the results of protein analysis using Thermo Scientific Proteome Discoverer 2.5 (San Jose, CA, USA) [17]. Functional analysis of the proteins in the result lists was performed using g:Profiler (https://biit.cs.ut.ee/gprofiler/gost) [20]. Protein interaction analysis of breed‐specific proteins was performed using STRING (https://string-db.org) [21].

## 3. Results and Discussion

**FIGURE 1:**
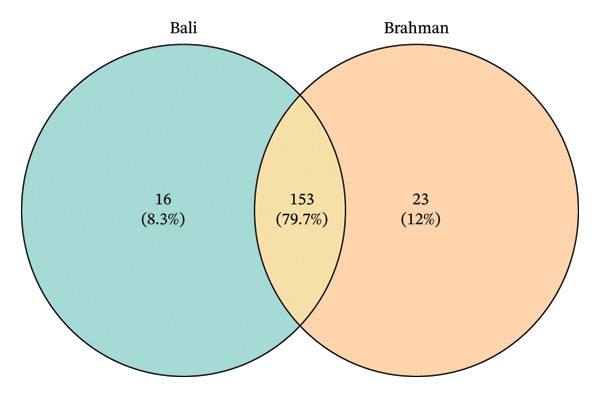
A Venn diagram showing the protein overlap between the Bali and Brahman cattle.

The Venn diagram showed a total of 192 proteins identified from the FF of both cattle breeds, of which 153 proteins (79.7%) were found as shared/core proteome, 16 proteins (8.3%) were unique in Bali cattle, and 23 proteins (12%) were unique in Brahman (Figure 1). The large proportion of shared proteins (> 75%) indicates that the basis of follicular physiology between breeds is relatively conservative, but the presence of unique proteins in each breed indicates specific molecular adaptations to different environmental conditions and reproductive physiology.

Proteomic analysis successfully identified several proteins that were expressed specifically in Bali and Brahman cattle. In Bali cattle, consistently detected proteins included ARMCX, AS3MT, CORO1C, RNF34, HEPACAM, L3MBTL3, IRAK1, LMBRD2, MEGF10, NDUFS7, LOC112442231, ZER1, RANGAP1, PTTG1, YARS1, and ZNF384, while in Brahman cattle, the dominant proteins included ACOX2, BHMT, CADM3, CDR2L, CLDN3, CCDC186, GABBR1, NFKBIL2, IF2D, KLHL13, KIAA0586, KIF13B, LCMT1, LOC535479, PRRT2, AGO1, PRKAR2B, SESN3, SLC9A8, SMARCE1, TMUB2, USP7, and ZNF132. These profiles indicate clear biological differences in the follicular microenvironment between the two breeds.

**FIGURE 2:**
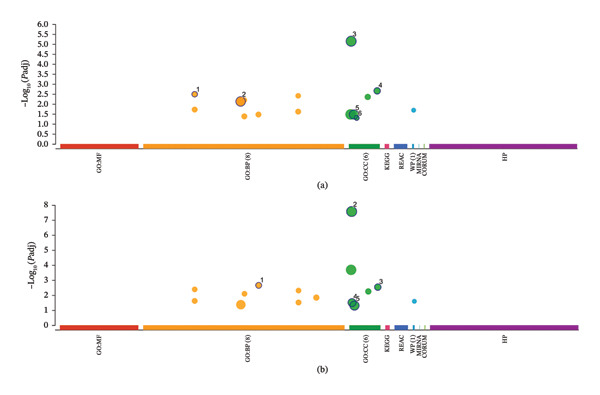
Bali’s (a) and Brahman’s (b) functional enrichment.

Gene Ontology (GO) (Table 1) and functional enrichment (Figure 2) showed that Bali cattle were prominent in the categories of negative regulation of blood coagulation, positive regulation of biological process, platelet alpha granule, and protease inhibitor‐related pathways (SERPINE2). Meanwhile, Brahman cattle exhibit enrichment in the lipid oxidation, peroxisomal metabolism, ATPase complex, and microtubule organizing center pathways, primarily influenced by the expression of ACOX2, BHMT, and KIAA0586. These findings are consistent with physiological differences between Bos indicus and Bos javanicus breeds in lipid metabolism and energy homeostasis.

**TABLE 1:** GO functional enrichment of Bali and Brahman cattle.

Source	Term ID	Term name	Padj	Protein
*Bali Cow*				
GO:BP	GO:0030195	Negative regulation of blood coagulation	3.237 × 10^−3^	ALOX12; FGG; FGB; SERPINE2; PLG
GO:BP	GO:0048518	Positive regulation of biological process	7.438 × 10^−3^	CORO1C; L3MBTL3; MEGF10; ZER1; YWHAH; APC; AJUBA; AHSG; A2M; APOA1; BMPER; CAMTA2; COLEC12; CUL7; DENND6A; CD248; FGG; FGB; SERPINE2; GCNT1; GOLGA4; GDF11; HAS3; INO80E; IRF4; KLF12; LIMK2; KIT; MED6; MTA3; MFHAS1; MYB; NOS2; DDX21; PCNT; PLG; RBPJ; ARHGAP35; TUT7; ATR; SCUBE3; MYORG; SKA3; SOX6; SYT4; TBX1; TBC1D7; TFPT; TEAD4; TPD52L1; ETV6; FGR; USP9X; ZNF804A
GO:CC	GO:0005737	Cytoplasm	7.125 × 10^−6^	CORO1C; RANGAP1; YARS1; YWHAH; CNP; APC; AJUBA; ALB; AHSG; APOA1; ALOX12; CPED1; CACNA2D1; CANX; CEPT1; CUL7; DENND6A; IGHMBP2; DNAJB6; DNAH7; EFCAB6; CD248; ELMO2; FASN; FCRLA; FGG; FGB; FIG4; SERPINE2; GCNT1; BPNT2; GOLGA4; GCC2; HECA; HAS3; IPO4; IRF4; KLF12; LIMK2; CAPG; MTA3; BOLA; SMAD9; CAD; MFHAS1; MYB; KMT5A; NOS2; DDX21; PARD6G; PCNT; EHHADH; LPIN1; PIP5K1B; GART; NDRG1; RBPJ; ARHGAP32; ARHGAP35; MARCHF8; TUT7; SEPTIN10; SHMT2; ATR; SDCCAG8; SORD; SNX11; MYORG; SKA3; SDHA; SYT4; TCAIM; TBC1D7; TEAD4; TSTD1; THOC1; TPD52L1; ETV6; TKTL1; TMEM199; TPRG1; FGR; USP9X; USH2A; VPS39; ZNF354C; ZNF804A
GO:CC	GO:1904949	ATPase complex	2.179 × 10^−3^	BICRAL; GATAD2B; INO80E; MTA3; DDX21; TFPT; TMEM199
GO:CC	GO:0012505	Endomembrane system	3.353 × 10^−2^	CORO1C; RANGAP1; APC; AJUBA; ALB; AHSG; CPED1; CACNA2D1; CANX; CEPT1; CUL7; DENND6A; FASN; FGG; FGB; FIG4; SERPINE2; GCNT1; BPNT2; GOLGA4; GCC2; HAS3; LEMD3; LIMK2; BOLA; LPIN1; NDRG1; ARHGAP32; MARCHF8; ATR; SNX11; MYORG; SYT4; TMEM199; VPS39; ZNF354C;
GO:CC	GO:0031091	Platelet alpha‐granule	4.978 × 10^−2^	FGG; FGB; SERPINE2

*Brahman Cows*				
GO:BP	GO:0061045	Negative regulation of wound healing	2.220 × 10^−3^	CLDN3; ALOX12; FGG; FGB; SERPINE2; PLG
GO:CC	GO:0005737	Cytoplasm	2.695 × 10^−8^	ACOX2; BHMT; CCDC186; GABBR1; KIF13B; LCMT1; PRRT2; AGO1; PRKAR2B; SESN3; SLC9A8; USP7; YWHAH; CNP; APC; AJUBA; ALB; AHSG; APOA1; ALOX12; CPED1; CACNA2D1; CANX; CUL7; DENND6A; IGHMBP2; DNAJB6; DNAH7; DTX3; EFCAB6; CD248; ELMO2; FASN; FCRLA; FGG; FGB; FIG4; SERPINE2; GCNT1; BPNT2; GOLGA4; GCC2; HECA; HAS3; IPO4; IRF4; KLF12; LIMK2; CAPG; MTA3; BOLA; SMAD9; CAD; MFHAS1; MYB; KMT5; ANOS2; DDX21; PARD6G; PCNT; EHHADH; LPIN1; PIP5K1B; GART; NDRG1; RBPJ; ARHGAP32; ARHGAP35; MARCHF8; TUT7; SEPTIN10; SHMT2; ATR; SDCCAG8; SORD; SNX11; MYORG; SKA3; SDHA; SYT4; TCAIM; TBC1D7; TEAD4; TSTD1; THOC1; TPD52L1; ETV6; TKTL1; TMEM199; TPRG1; FGR; USP9X; USH2A; VPS39; ZNF354C; ZNF804A
GO:CC	GO:1904949	ATPase complex	2.918 × 10^−3^	BICRAL; GATAD2B; INO80E; MTA3; DDX21; TFPT; TMEM199
GO:CC	GO:0005815	Microtubule organizing center	3.097 × 10^−2^	KIAA0586; PRKAR2B; AHI1; APC; CUL7; LIMK2; CAPG; PCNT; NDRG1; ARHGAP35; SDCCAG8; SKA3; TBC1D7; USP9X; USH2A
GO:CC	GO:0012505	Endomembrane system	4.867 × 10^−2^	CCDC186; PRRT2; SLC9A8; APC; AJUBA; ALB; AHSG; CPED1; CACNA2D1; CANX; CEPT1; CUL7; DENND6A; FASN; FGG; FGB; FIG4; SERPINE2; GCNT1; BPNT2; GOLGA4; GCC2; HAS3; LEMD3; LIMK2; BOLA; LPIN1; NDRG1; ARHGAP32; MARCHF8; ATR; SNX11; MYORG; SYT4; TMEM199; VPS39; ZNF354C

**FIGURE 3:**
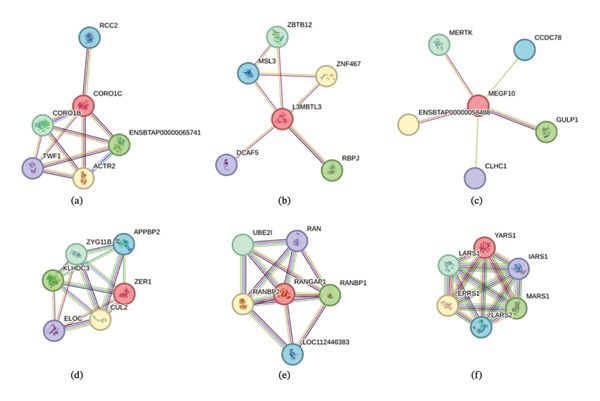
Breed‐specific proteins signatures of follicular physiology in Bali cow. (a) CORO1C. (b) L3MBTL3. (c) MEGF10. (d) ZER1. (e) RANGAP1. (f) YARS1.

STRING analysis detailed the interaction network for FF‐specific proteins, revealing key functional and structural relationships. In Bali cows, six major protein clusters identified through STRING (Figure 3) and functional enrichment analysis showed involvement in cytoskeletal remodeling, epigenetic regulation, phagocytosis, ubiquitination, nucleocytoplasmic transport, and aminoacyl‐tRNA biosynthesis.

The first prominent cluster consists of CORO1C, CORO1B, ACTR2, TWF1, and RCC2, which represents the actin cytoskeleton dynamics module. CORO1C and CORO1B are members of the coronin family that act as regulators of F‐actin binding and control the activity of the Arp2/3 complex through ACTR2, thereby regulating branched actin polymerization [22, 23]. TWF1 functions in actin turnover by binding actin monomers, enabling structural plasticity of the cell [24]. The interaction of RCC2 within this cluster indicates a link between actin reorganization and cell cycle regulation, particularly during cell division and migration [25]. In a reproductive context, this module is highly relevant to oocyte maturation, cumulus expansion, and early embryonic cleavage, which are highly dependent on actin dynamics [26].

The second cluster centers on L3MBTL3, which interacts with MSL3, ZBTB12, ZNF467, RBPJ, and DCAF5, reflecting a module for chromatin regulation and epigenetic transcriptional control. L3MBTL3 is a histone methylation reader protein that plays a role in chromatin regressivity and regulating cell differentiation [27]. Its interaction with MSL3 links this cluster to the regulation of histone H4K16 acetylation, a key epigenetic modification in the control of gene expression [28]. The presence of RBPJ, a key mediator of the Notch pathway, suggests a link between epigenetic regulation and cell differentiation signals [29]. In ovarian follicles, notch signaling has been implicated in granulosa cell proliferation, follicular development, and oocyte–somatic cell communication [30]. The tight interaction among these proteins indicates coordinated transcriptional and epigenetic regulation critical for maintaining cellular functionality. This module is highly relevant in determining the developmental competence of germ cells and embryos, where precise epigenetic regulation is a determinant of embryo quality.

Another identified cluster center on MEGF10, which interacts with MERTK, GULP1, CCDC78, and CLHC1, represents a module for phagocytosis and apoptotic cell clearance. MEGF10 and MERTK are phagocytic receptors involved in the recognition and elimination of dead cells [31]. The interaction with the adaptor GULP1 confirms the role of this cluster in the internalization and degradation of cellular debris. In reproductive tissues, efficient phagocytosis is crucial for ovarian tissue homeostasis and the selection of apoptotic cells, thus contributing to the quality of the follicular microenvironment [32]. MERTK is a receptor tyrosine kinase essential for recognizing apoptotic cells and suppressing excessive inflammatory responses [31].

The CUL2, ELOB, ZYG11B, KLHDC3, APPBP2, and ZER1 clusters represent a module of the ubiquitin ligase complex (Cullin‐RING ligase) involved in controlled protein degradation. The CUL2 complex functions as a key scaffold in the ubiquitination of target proteins, regulating the stability of proteins involved in the cell cycle and differentiation [33]. In reproductive cells, the ubiquitin–proteasome system is a crucial mechanism for protein quality control during oocyte maturation and early embryonic development.

The RAN, RANBP1, RANBP2, RANGAP1, and UBE2I cluster represents a nucleocytoplasmic transport module. The RAN GTPase system regulates protein and RNA transport across the nuclear membrane and mitotic spindle dynamics [34]. This module is crucial during meiotic transition and embryonic cleavage, where the nucleocytoplasmic distribution of proteins must be tightly controlled. The final, highly dense cluster consists of LARS1, IARS1, MARS1, YARS1, EPRS1, and LARS2, which represent aminoacyl‐tRNA synthetases (aaRSs), essential enzymes in protein translation. In addition to their canonical functions, aaRSs are known to have noncanonical roles in the regulation of cell signaling, stress, and differentiation [35]. Efficient translational activity is crucial during oocyte maturation and early embryonic development, when rapid and coordinated protein synthesis is required [36].

**FIGURE 4:**
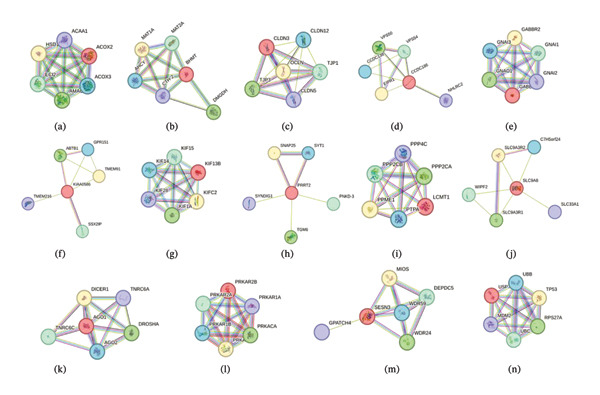
Breed‐specific proteins signatures of follicular physiology in Brahman cow. (a) ACOX2. (b) BHMT. (c) CLDN3. (d) CCDC186. (e) GABBR1. (f) KIAA0586. (g) KIF13B. (h) PRRT2. (i) LCMT1. (j) SLC9AB. (k) AGO1. (l) PRKAR2B. (m) SESN3. (n) USP7.

The protein–protein interaction network image of Brahman cow (Figure 4) reveals several distinct but highly organized protein clusters, each representing a specific biological functional module. This pattern suggests that the detected proteins do not operate randomly but are instead involved in coordinated biological pathways encompassing energy metabolism, signal transduction regulation, epithelial integrity, intracellular transport, protein quality control, and posttranscriptional regulation of gene expressions.

One major cluster consists of ACAA1, ACOX2, ACOX3, AMACR, ECI2, and HSD17B4, which are key components of peroxisomal fatty acid metabolism, particularly β‐oxidation of very long‐chain fatty acids. This pathway plays a crucial role in cellular energy homeostasis and the control of oxidative stress [37, 38]. In the context of reproduction and cell physiology, peroxisomal activity contributes to germ cell quality and the cellular microenvironment through the regulation of bioactive lipids and cellular redox [39]. Another prominent cluster is the one‐carbon metabolism module involving MAT1A, MAT2A, BHMT, AHCY, and GNMT. This pathway plays a role in the biosynthesis of S‐adenosylmethionine (SAM), a universal methyl donor essential for DNA, RNA, and protein methylation. Regulation of one‐carbon metabolism is crucial for epigenetic control during cell differentiation and early embryonic development [40]. Dysfunction of this module has been linked to impaired oocyte competence and mammalian embryonic development.

The tight junction cluster, consisting of CLDN3, CLDN5, CLDN12, OCLN, TJP1, and TJP3, reflects the crucial role these proteins play in epithelial barrier integrity and intercellular communication. Tight junctions function not only as a physical barrier but also as a signal transduction platform that regulates cell polarity and proliferation [41]. In reproductive tissues, tight junctions play a crucial role in maintaining follicular homeostasis and regulating the transport of small molecules that influence oocyte maturation [42].

The transport protein module and cytoskeleton dynamics are represented by the KIF1A, KIF3B, KIF14, KIF15, KIF28, and KIFC2 clusters, which are members of the kinesin family. These proteins play a role in vesicular transport, chromosome segregation, and spindle formation during cell division [43]. Kinesin activity is crucial in oocyte meiosis and early embryonic cleavage, where impaired motor protein function can lead to aneuploidy and embryonic developmental failure [44].

The PPP2CA, PPP2CB, PPP4C, PPME1, LCMT1, and PTPA clusters represent the protein phosphatase 2A (PP2A) module, a central regulator of protein phosphorylation. PP2A controls various critical processes such as the cell cycle, apoptosis, and signal transduction [45]. In reproductive cells, PP2A plays a role in regulating meiotic maturation and spindle stability, making it a crucial determinant of oocyte quality [46]. The network also displays the DICER1, DROSHA, AGO1, AGO2, TNRC6A, and TNRC6C clusters, which reflect the microRNA (miRNA) processing machinery. This system plays a role in posttranscriptional regulation of gene expression and has been shown to be essential for oocyte development, embryo implantation, and cellular adaptation to physiological stress [47, 48]. Dysregulation of miRNAs is frequently associated with decreased fertility and embryonic developmental failure.

The PRKACA, PRKAR1A, PRKAR1B, PRKAR2A, and PRKAR2B clusters suggest the involvement of protein kinase A (PKA) signaling, a key cAMP‐mediated pathway. PKA plays a role in regulating oocyte maturation, hormonal responses, and cell metabolism [49]. Precise activation and inhibition of PKA are required for meiotic transition and oocyte cytoplasmic maturation. Furthermore, the TP53, MDM2, USP7, UBC, and RPS27A clusters reflect a protein quality control module and cellular stress response via the p53–ubiquitin pathway. This system maintains genome stability and determines cell fate under stress conditions [50]. In the reproductive system, the p53 pathway plays a role in oocyte and embryo quality selection, ensuring that only cells with optimal genomic integrity can develop further [51].

Differences in proteomic composition between Bali and Brahman cattle reflect biological variations in the follicular microenvironment. In Bali cattle, CORO1C expression is an important finding because this protein plays a role in the dynamics of the F‐actin cytoskeleton, which is essential for meiotic spindle formation and oocyte cytoplasmic reorganization. Recent studies have shown that actin‐regulating proteins can influence oocyte quality and early embryonic development [52]. Proteins such as FGG, FGB, and SERPINE2 also reinforce the Balinese profile, suggesting involvement in extracellular matrix remodeling and protease regulation, processes necessary for the maturation and expansion of cumulus cells. The role of SERPINE2 in granulosa function has been reported as a biomarker of oocyte competence [53].

In Brahman cattle, the presence of BHMT reflects the dominance of the one‐carbon metabolism (1C) pathway, which is closely linked to methionine homeostasis and oocyte DNA methylation. Variations in 1C metabolism have been shown to influence oocyte quality across species [52]. Meanwhile, ACOX2 expression indicates peroxisomal β‐oxidation activity, which is crucial for maintaining lipid metabolism balance and reducing oxidative stress in the follicular microenvironment [54]. These differences suggest that although MII levels are not significantly different, the molecular factors regulating oocyte competence may differ between breeds. Brahman cattle appear to have a follicular environment with more active lipid metabolism, while Bali cattle have a follicular environment more influenced by protease regulation and cytoskeleton dynamics.

These findings align with previous FF proteomics literature, which shows that FF protein composition is closely correlated with oocyte competence and successful maturation, fertilization, and embryo development [55, 56]. Furthermore, differences in proteomic composition between cattle breeds have previously been reported to be influenced by genetic, physiological, and metabolic factors, such as negative energy balance, which affects FF and oocyte quality [57]. Homocysteine imbalance in FF in other species has been shown to reduce oocyte quality [52]. Therefore, the proteomic differences found could be used as candidate breed‐specific biomarkers for oocyte quality. However, this remains speculative and requires further functional validation accompanied by reproductive outcome evaluation data such as oocyte competence, embryo development, conception rate, or pregnancy success.

## 4. Conclusion

In this study, we compared specific proteins in FF from the ovaries of Bali and Brahman cattle. Notably, we identified several bioactive proteins as candidate proteins potentially associated with reproductive processes, although further validation is still needed with the support of reproductive outcome. This study provides a foundation for further research on cattle breeding and genetic resource management, contributing to the sustainable development of livestock farming in tropical countries.

## Funding

The authors are grateful to the Ministry of Education, Culture, Research, and Technology of the Republic Indonesia for funding the research with grant number: 02209/UN4.22/PT.01.03/2025.

## Conflicts of Interest

The authors declare no conflicts of interest.

## Data Availability

The data supporting this study’s findings can be obtained from the corresponding author upon a reasonable request.
